# Spatial and temporal gene expression patterns during early human odontogenesis process

**DOI:** 10.3389/fbioe.2024.1437426

**Published:** 2024-07-16

**Authors:** Yejia Yu, Kun Wang, Zhuo Wang, Haoyang Cai, Chengcheng Liao, Yutao Wu, Jingyi Zhang, Weidong Tian, Li Liao

**Affiliations:** ^1^ State Key Laboratory of Oral Diseases and National Clinical Research Center for Oral Diseases and Engineering Research Center of Oral Translational Medicine, Ministry of Education and National Engineering Laboratory for Oral Regenerative Medicine, West China Hospital of Stomatology, Sichuan University, Chengdu, China; ^2^ Center of Growth, Metabolism and Aging, Key Laboratory of Bio-Resource and Eco-Environment of Ministry of Education, College of Life Sciences, Sichuan University, Chengdu, China; ^3^ Chengdu Shiliankangjian Biotechnology Co., Ltd., Chengdu, China

**Keywords:** laser-capture microdissection, RNA sequencing, odontogenesis, human deciduous tooth germ, species comparison

## Abstract

Studies on odontogenesis are of great importance to treat dental abnormalities and tooth loss. However, the odontogenesis process was poorly studied in humans, especially at the early developmental stages. Here, we combined RNA sequencing (RNA-seq) with Laser-capture microdissection (LCM) to establish a spatiotemporal transcriptomic investigation for human deciduous tooth germs at the crucial developmental stage to offer new perspectives to understand tooth development and instruct tooth regeneration. Several hallmark events, including angiogenesis, ossification, axonogenesis, and extracellular matrix (ECM) organization, were identified during odontogenesis in human dental epithelium and mesenchyme from the cap stage to the early bell stage. ECM played an essential role in the shift of tooth-inductive capability. Species comparisons demonstrated these hallmark events both in humans and mice. This study reveals the hallmark events during odontogenesis, enriching the transcriptomic research on human tooth development at the early stage.

## Graphical Abstract

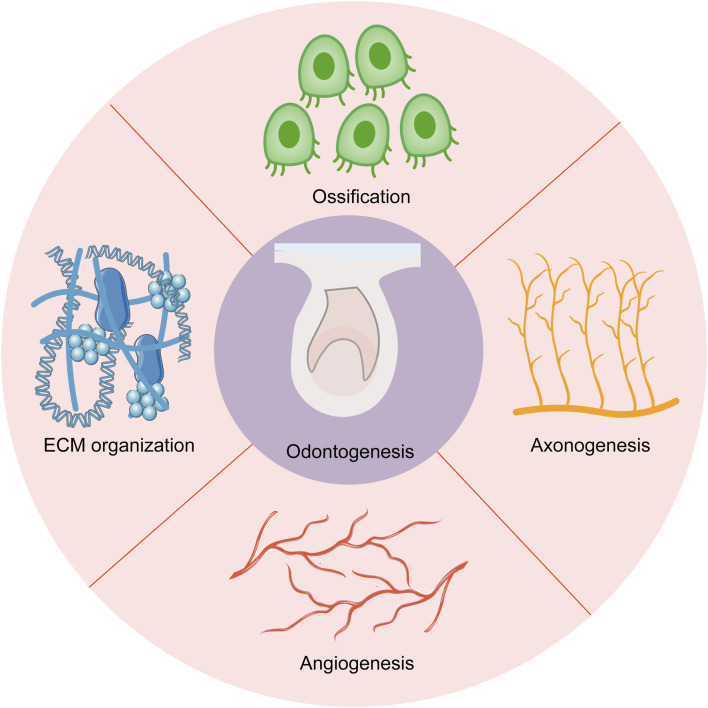

## 1 Introduction

Human tooth development starts from the embryonic period, which involves a complex sequence of reciprocal interactions between dental epithelium and neural crest-derived mesenchyme (Kollar and Baird, 1970). The development of tooth germ goes through three distinct morphological changes (Bud stage, Cap stage, and Bell stage), in which several signaling pathways, like Sonic Hedgehog Signaling Molecule (SHH), Fibroblast Growth Factor (FGF), Bone Morphogenetic Protein (BMP), and Wingless-Type MMTV Integration Site Family (WNT), are activated in an orderly manner between dental epithelium and dental mesenchyme (Thesleff and Sharpe, 1997; Alghadeer et al., 2023). Based on murine models, numerous growth factors including BMP4, SHH, WNT, and FGF8, as well as transcription factors like Msh Homeobox 1 (*MSX1*)*,* Paired Box 9 (*PAX9*)*,* and LIM Homeobox 6 (*LHX6*), have been identified as necessary signaling molecules in tooth pattern formation (Vainio et al., 1993; Satokata and Maas, 1994; Peters et al., 1998; Trumpp et al., 1999; Zhang et al., 2005). The spatial-temporal expression of transcription factors and growth factors forms a complex and delicate signaling network to regulate odontogenesis. Odontogenic potential, also known as tooth-inductive capability, can induce specific gene expression in adjacent tissues and thus instruct tooth formation (Hu et al., 2014). A series of mouse tooth germ combination assays have demonstrated that odontogenic potential resides initially in dental epithelium and then shifts to dental mesenchyme after E12.5 (Mina and Kollar, 1987; Lumsden, 1988).

Although previous studies on mouse molar models provide valuable insights into human tooth formation patterns, the differences between the mouse and human tooth formation should not be dismissed, as the cell types, the shape of the crown, the number of roots, and the shift of odontogenic potential between the epithelium and mesenchyme varying between the two species (Kavanagh et al., 2007; Krivanek et al., 2020). Unlike murine teeth, Human fetus tooth germ recombination experiments have identified that dental epithelium from the cap stage (DE-cap) and dental mesenchyme from the bell stage (DM-bell) possess tooth-inductive capability (Hu et al., 2014), which highlighted the role of the cap stage and the early bell stage during the odontogenesis process in humans.

Chen et al. (2023) have collected dental epithelium and mesenchyme from mouse E11.5 and E13.5 embryos, revealing the roles of extracellular proteoglycans in mouse tooth development at the crucial developmental stage when the odontogenic potential shift occurs by RNA sequencing (RNA-seq). Due to the challenges in obtaining human embryo samples, as well as the indistinct demarcation between the tooth germs and the surrounding tissues at the early embryonic stage (Alghadeer et al., 2023), the dynamic gene expressions in human tooth germs remain elusive. Laser-capture microdissection (LCM) is a reliable technique to obtain pure and precise populations of target cells under microscopic visualization (Bonner et al., 1997). We therefore sought to combine RNA-seq with LCM of human tooth germs at the cap and the early bell stage, to provide a relatively comprehensive and tissue-specific transcriptomic investigation of the tooth formation process without losing the native spatial information. These data revealed characteristic transcriptomes of the dental epithelium and dental mesenchyme at different developmental stages, and provided insights into crucial signaling pathways and genes involved in odontogenesis.

## 2 Materials and methods

### 2.1 Tissue collection, embedding and sectioning

The study was conducted in accordance with the Declaration of Helsinki, and approved by the Institutional Review Board of West China Second University Hospital, Sichuan University (2022-307). Human embryos from voluntary termination of pregnancy surgery were collected from West China Second University Hospital, Sichuan University under informed consent from donors, excluding donors with chronic diseases, infectious diseases, hereditary disorders, or abnormal pregnancies. All samples were collected via surgical abortion, and embryos subjected to medication-induced abortion were excluded from this study. The age of aborted embryos was measured in weeks from the first day of the donor’s last menstrual cycle to the day when it was aborted, which was reconfirmed by ultrasound examination.

The samples were transferred to the laboratory in RNase-free PBS on ice within 2 h. Mandibles were dissected from fetal craniofacial tissues under a stereomicroscope (Olympus). All the reagents and instruments were treated with RNase-removing reagents (Vazyme). The fresh tissues were washed three times with cold RNase-free PBS and embedded in optimum cutting temperature (OCT) compound, then the embedded samples were transferred to −80°C for at least 1 h before sectioning. The sections (10 μm) were prepared in a cryostat at −20°C and carefully collected by UV light-treated MMI Membrane Slides (Molecular Machines & Industries).

### 2.2 Slices staining

Different concentrations of ethanol solutions were prepared from 100% (vol/vol) ethanol and nuclease-free H_2_O. The slices were put into 100% (vol/vol) ethanol for 30 s and 75% (vol/vol) ethanol for 1–3 min, then stained with 1% (wt/vol) cresyl violet for 1 min. Subsequently, slides were washed in DEPC H_2_O and dehydrated in 75%, 100% (vol/vol) ethanol solutions for 30 s. Last, the slices were placed at room temperature for 1-2 min until they were dry.

### 2.3 Laser microdissection

Samples were collected by laser microdissection as previously described (Nichterwitz et al., 2018). Before dissection, we put a cover-glass slide under the stained slide to make a sandwich. The sandwiches were placed into the slide holder of the microscope (Nikon) and images were captured by MMI CellCut Plus system (Molecular Machines & Industries). Laser thickness, power parameters, and laser spot were adjusted before collection. Cutting outlines were drawn closely around the interest areas at 100X magnification, and the cut tissues were collected in diffuser caps (Molecular Machines & Industries).

### 2.4 cDNA and sequencing library preparation

A protocol developed by Chen et al. (2017) was followed in this part. Briefly, the collected samples were lysed with 50 μL of 4 m guanidine isothiocyanate (GuSCN) solution and incubated at 42°C for 20 min, then they were transferred to 1.5 mL microcentrifuge tubes and mixed with glycogen precipitating buffer at −80°C for at least 1 h. After being concentrated and washed in the 75% ethanol solution, the RNA molecules were reverse-transcribed and amplified with a modified Smart-seq2 single-cell-based PCR technique. Sequencing was performed on an Illumina Novaseq 6,000 platform.

### 2.5 Human RNA-seq data analysis

Quality control of raw data using fastqc, and processing of adaptors and low-quality reads using trim_galore (version 0.6.4) to get clean reads. Then the clean reads were mapped against the human genome (GRCh38/hg38) by using hisat2 (version 2.2.0). Expression levels of each gene were quantified using featureCounts (version 1.5.3) with the reference annotation (*Homo Sapiens*, GRCh38.108) (Liao et al., 2014). Removing genes with a sum of expression less than 20 in each row to obtain a high-quality expression matrix. And genes differential expression analysis was performed by DESeq2 (version 1.42.0) (Love et al., 2014). The differentially expressed genes were rigorously analyzed using a stringent cutoff criterion of a log fold change greater than 2 and an adjusted *p*-value less than 0.05. Then these genes were used for enrichment analysis with clusterProfiler (version 4.11.0) and ReactomePA (version 1.16.0) (Yu and He, 2016; Wu et al., 2021). Spatial mapping of marker genes was done using the DistMap method (Pandey and Schlötterer, 2013). The input data which contains immunohistochemistry, immunofluorescence, and in-situ hybridization data of dental marker genes (in total 103 genes used in E14.5 spatial mapping) was from Hu et al. (2022). The source code was freely avaliable through GitHub (https://github.com/victorwang123/RNASeq-Processing).

### 2.6 Mouse RNA-seq data analysis

Expression matrix was downloaded from the NCBI Gene Expression Omnibus (GEO) <GSE209968>. The differentially expressed genes were analyzed using DESeq2 package (version 1.42.0) by the cutoff of log fold change >2 and P adjusted value <0.05 (Love et al., 2014). Functional annotation and enrichment analysis of the significantly differentially expressed genes were performed with clusterProfiler package (version 4.11.0) (Wu et al., 2021).

### 2.7 Immunofluorescence staining

Fetal mandible segments were fixed in 4% paraformaldehyde overnight at 4°C. Samples were embedded in paraffin and sectioned into 5 μm slices. The sections were deparaffinized, dehydrated, and boiled in a pressure cooker in citric acid buffer (pH = 6.0) for 20 min for antigen retrieval. Then, they were blocked by 10% donkey serum (Solarbio) for 1 h at room temperature. Primary antibodies, including VEGF (abcam), CD31 (abcam), GAP43 (zenbio), and Tubulin β III (abcam), were diluted into 1:100 and incubated overnight at 4°C. Both secondary antibodies (Invitrogen) and DAPI (Solarbio) at 1:500 dilution were applied for 1 h and 5 min respectively at room temperature. Autofluorescence was quenched according to the manufacturer’s instructions (Vector). Images were captured by the Olympus system.

## 3 Results

### 3.1 Transcriptional profiling of the dental epithelium and mesenchyme revealed cell type-specific gene expression

**FIGURE 1:**
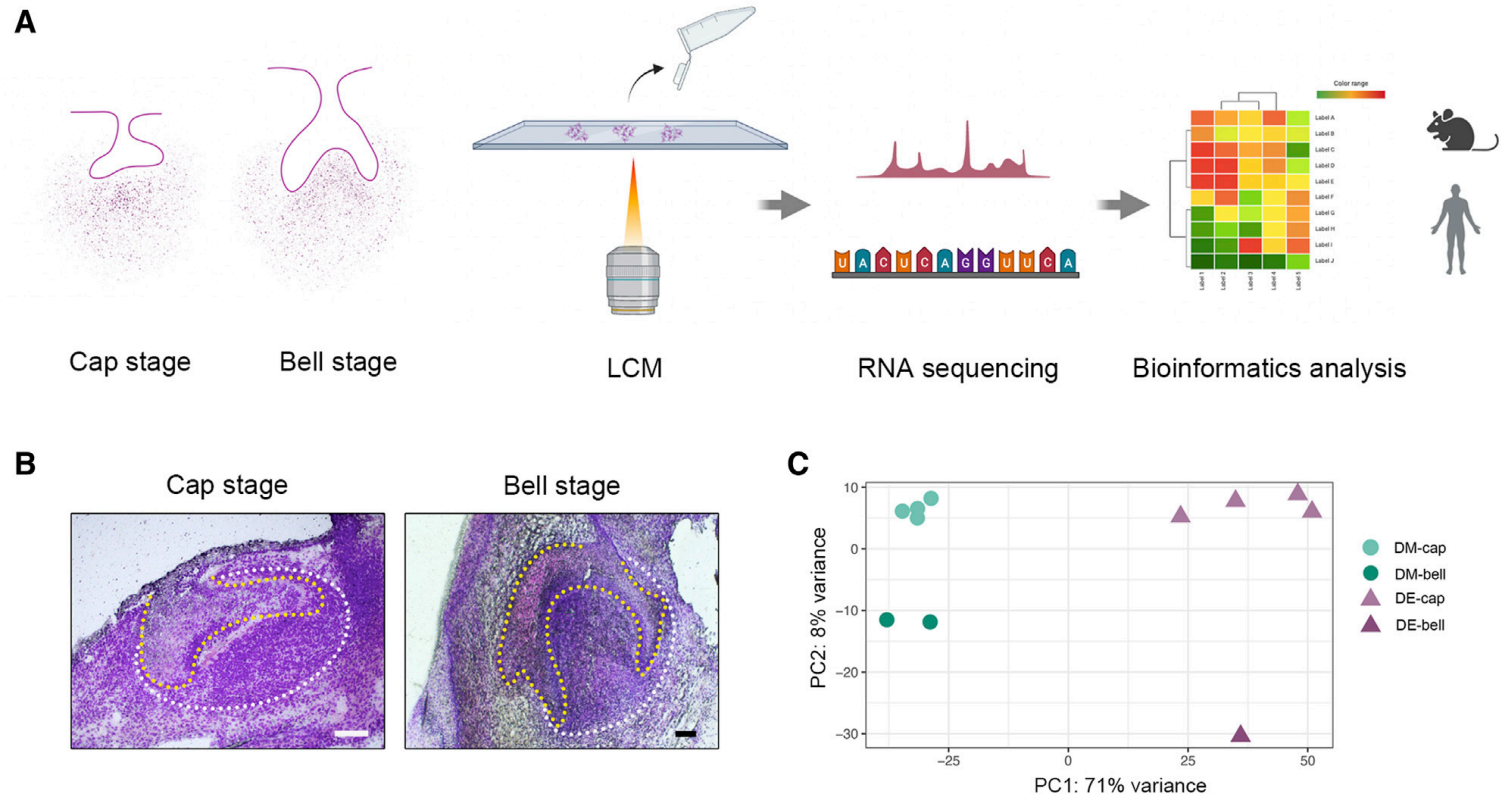
LCM strategy to reveal spatial and temporal gene expression patterns during early human odontogenesis process. **(A)** Schematic of the experimental workflow. We collected human deciduous tooth germs at the cap and early bell stage. Then, we isolated dental epithelium and dental mesenchyme by LCM. RNA-seq was combined with LCM to generate a spatial-temporal transcriptomic profile of odontogenesis in human beings. **(B)** Human tooth germs at the cap stage and early bell stage before LCM. The yellow line outlines dental epithelium; the white line outlines dental mesenchyme. Scale bar: 60 μm. **(C)** Principal component analysis (PCA) on the whole gene expression data set. DM-cap: dental mesenchyme at the cap stage; DM-bell: dental mesenchyme at the early bell stage; DE-cap: dental epithelium at the cap stage; DE-bell: dental epithelium at the early bell stage.

Previous studies have identified that tooth-inductive capability shifts from dental epithelium to dental mesenchyme at the early bell stage in human tooth development (Hu et al., 2014). To better investigate the underlying mechanisms, we collected different types of human embryo deciduous tooth germs from the gestational week (GW) 8 to GW13, which was the crucial period when the shift occurred. Collectively, we obtained four deciduous tooth germs at the cap stage and two deciduous tooth germs at the early bell stage. LCM was applied to isolate dental epithelium and dental mesenchyme tissues from tooth germs, which coupled with RNA sequencing offered us a library of dental epithelium and dental mesenchyme at different developmental stages (Figures 1A, B).

After conducting laser-capture microdissection and RNA sequencing (LCM-seq), we performed quality control, using only samples in which we achieved a gene detection level of greater than 11,000 expressed genes, which left a total of six dental epithelium samples (four from the cap stage and two from the early bell stage) and five dental mesenchyme samples (four from the cap stage and one from the early bell stage) for further analysis. Principal component analysis (PCA) on the entire gene expression data set revealed the tissue heterogeneity between dental epithelium and dental mesenchyme (Figure 1C).

**FIGURE 2:**
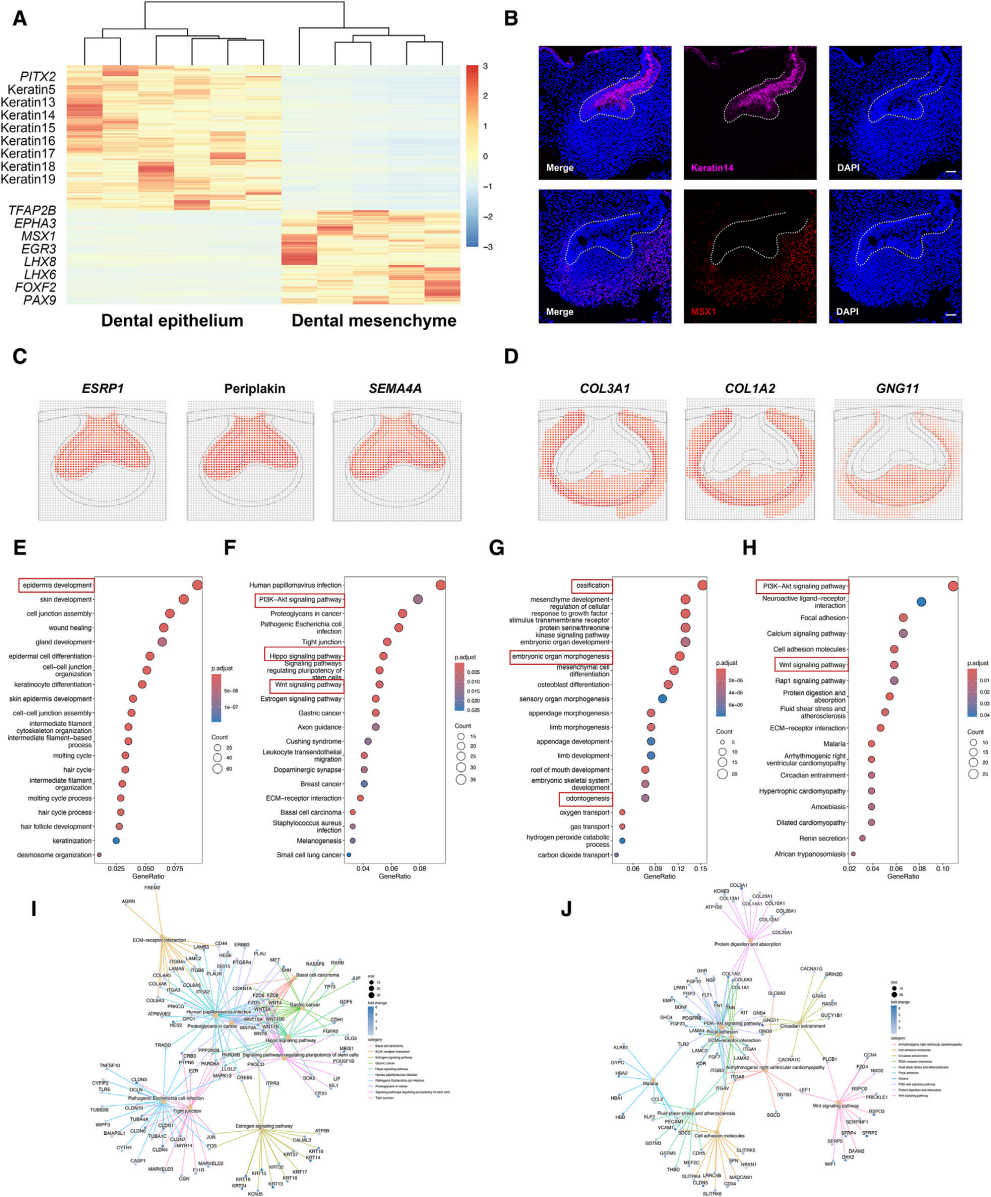
Cell type-specific gene expression between dental epithelium and dental mesenchyme. **(A)** Heatmap for DEGs identified in dental epithelium and dental mesenchyme. **(B)** Representative immunofluorescence staining images of dental epithelium and dental mesenchyme. The white line outlines dental epithelium. Scale bar: 50 μm. **(C)** DistMap results for the predicted markers in dental epithelium. **(D)** DistMap results for the predicted markers in dental mesenchyme. **(E)** GO analysis for DEGs upregulated in dental epithelium. **(F,I)** KEGG pathway analysis for DEGs upregulated in dental epithelium. **(G)** GO analysis for DEGs upregulated in dental mesenchyme. **(H,J)** KEGG pathway analysis for DEGs upregulated in dental mesenchyme.

In-depth gene expression analysis across dental epithelium and dental mesenchyme confirmed known marker gene expression of the two types of tissues, including Paired Like Homeodomain 2 (*PITX2*), Keratin5, Keratin14, and Keratin17 detected in dental epithelium, as well as Transcription Factor AP-2 Beta (*TFAP2B*)*, MSX1, LHX8, LHX6,* and *PAX9* detected in dental mesenchyme (Figure 2A). The immunofluorescence staining for PITX2 and MSX1 further confirmed the expression patterns of specific markers in the dental epithelium and mesenchyme (Figure 2B). Besides, several novel cell type-specific transcripts showed in differentially expressed genes (DEGs) were identified by DistMap, including Epithelial Splicing Regulatory Protein 1 (*ESRP1*)*,* Periplakin, and Semaphorin 4A (*SEMA4A*) expressed in dental epithelium, as well as Collagen Type III Alpha 1 Chain (*COL3A1*)*, COL1A2,* and G Protein Subunit Gamma 11 (*GNG11*) expressed in dental mesenchyme, suggesting their spatial distribution and cell-type specific characteristics (Figures 2C, D).

In dental epithelium, Gene ontology (GO) analysis of DEGs mostly enriched epidermis development (Figure 2E). Kyoto Encyclopedia of Genes and Genomes (KEGG) pathway analysis showed that PI3K-Akt, Hippo, and Wnt signaling pathways were activated in dental epithelium (Figures 2F, I). As to dental mesenchyme, GO analysis of DEGs enriched several biological processes, including ossification, embryonic organ morphogenesis, and odontogenesis (Figure 2G). KEGG pathway analysis revealed that PI3K-Akt signaling pathway, Wnt signaling pathway, Focal adhesion, and extracellular matrix (ECM)-receptor interaction were enriched in dental mesenchyme (Figures 2H, J).

Collectively, the dental epithelium and dental mesenchyme exhibited distinct gene expression profiles. The biological processes and signaling pathways associated with the dental epithelium and dental mesenchyme closely corresponded to the tooth formation process. This suggests that samples isolated using LCM were of high quality and accuracy, making them suitable for further analysis.

### 3.2 Temporal transcriptional changes in the dental epithelium before and after the shift in odontogenic potential

**FIGURE 3:**
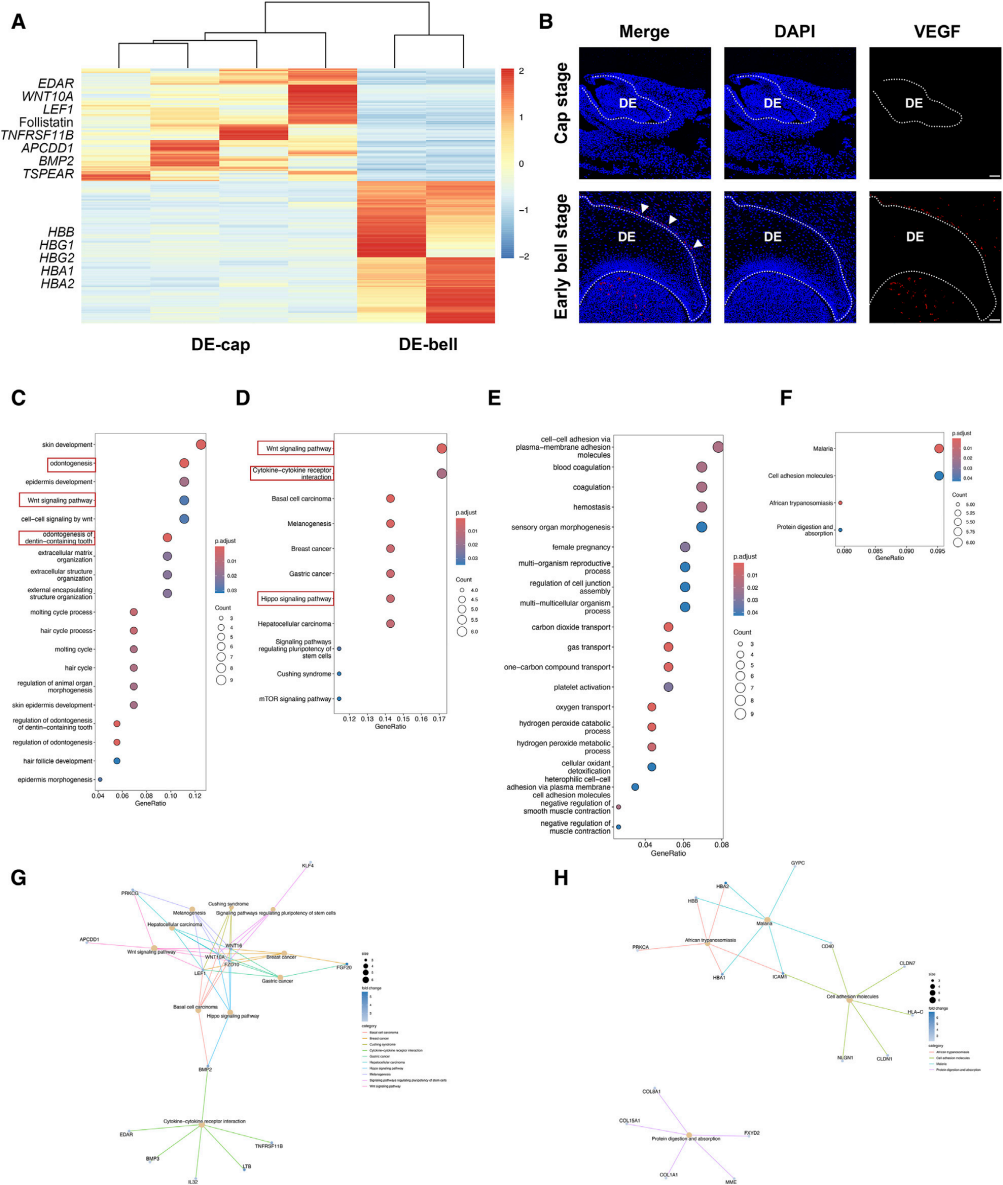
Temporal transcriptional changes between DE-cap and DE-bell. **(A)** Heatmap for DEGs identified in DE-cap and DE-bell. **(B)** Representative immunofluorescence staining images of VEGF in DE-cap and DE-bell. The white line outlines dental epithelium. DE: dental epithelium. Scale bar: 50 μm. **(C)** GO analysis for DEGs upregulated in DE-cap. **(D,G)** KEGG pathway analysis for DEGs upregulated in DE-cap. **(E)** GO analysis for DEGs upregulated in DE-bell. **(F,H)** KEGG pathway analysis for DEGs upregulated in DE-bell.

The tooth-inductive capacity resides in the dental epithelium at the cap stage (DE-cap), while it shifts to dental mesenchyme at the early bell stage (DM-bell). To figure out the dynamic transcriptional changes residing in dental epithelium before and after the odontogenic potential shift, we compared gene expression patterns in dental epithelium between the cap stage and the early bell stage. A total of 119 DEGs were identified in DE-cap, including several known odontogenesis-related markers, such as Ectodysplasin A Receptor (*EDAR*)*, WNT10A,* Lymphoid Enhancer Binding Factor 1 (*LEF1*)*,* Follistatin, TNF Receptor Superfamily Member 11b (*TNFRSF11B*)*,* APC Downregulated 1 (*APCDD1*)*, BMP2,* and Thrombospondin Type Laminin G Domain And EAR Repeats (*TSPEAR*) (Figure 3A). GO analysis of these DEGs pinpointed a number of biological processes related to tooth formation, like odontogenesis, Wnt signaling pathway, and odontogenesis of dentin-containing tooth (Figure 3C). KEGG pathway analysis showed that odontogenesis-related signaling pathways like Wnt, Hippo, and cytokine-cytokine receptor interaction were activated in DE-cap (Figures 3D, G).

As to dental epithelium at the early bell stage (DE-bell), we identified 129 DEGs in total. GO analysis revealed a large number of biological processes associated with erythrocytes, suggesting extensive angiogenesis activities in DE-bell. The upregulated expression of hemoglobin-related markers, such as Hemoglobin Subunit Beta (*HBB*)*,* Hemoglobin Subunit Gamma 1 (*HBG1*)*, HBG2,* Hemoglobin Subunit Alpha1 (*HBA1*)*,* and *HBA2,* were enriched in gas transport and oxygen transport (Figure 3E). KEGG pathway analysis did not show odontogenesis-related signaling pathways (Figures 3F, H). Vascular Endothelial Growth Factor A (VEGF) is a key factor participating in angiogenesis, vasculogenesis and endothelial cell growth (Apte et al., 2019). CD31, also known as platelet endothelial cell adhesion molecule 1 (PECAM-1), is thought to be a sensitive and specific marker for endothelial cells. Immunofluorescence staining of VEGF and CD31 revealed abundant blood vessels intricately surrounding the outer enamel epithelium at the early bell stage. Additionally, blood vessels were detected in the dental papilla at this developmental stage (Figure 3B; Supplementary Figure S1).

In summary, the transition from DE-cap to DE-bell involved the loss of odontogenic capacity and the development of blood vessels surrounding the outer enamel epithelium. In fact, immunofluorescence staining of VEGF and CD31 revealed extensive angiogenesis throughout the entire tooth germ at the early bell stage. The tooth germ progresses into a mature phase at the bell stage, during which odontoblasts and ameloblasts begin differentiation and function. Extensive angiogenesis facilitates the provision of ample oxygen and nutrient supply to the developing organism (Crivellato, 2011). We hypothesized that although DE-bell lost odontogenic capacity, the newly formed blood vessels in DE-bell and dental papilla played essential roles in the odontogenesis process at the early bell stage.

### 3.3 Temporal transcriptional changes in the dental mesenchyme before and after the shift in odontogenic potential

**FIGURE 4:**
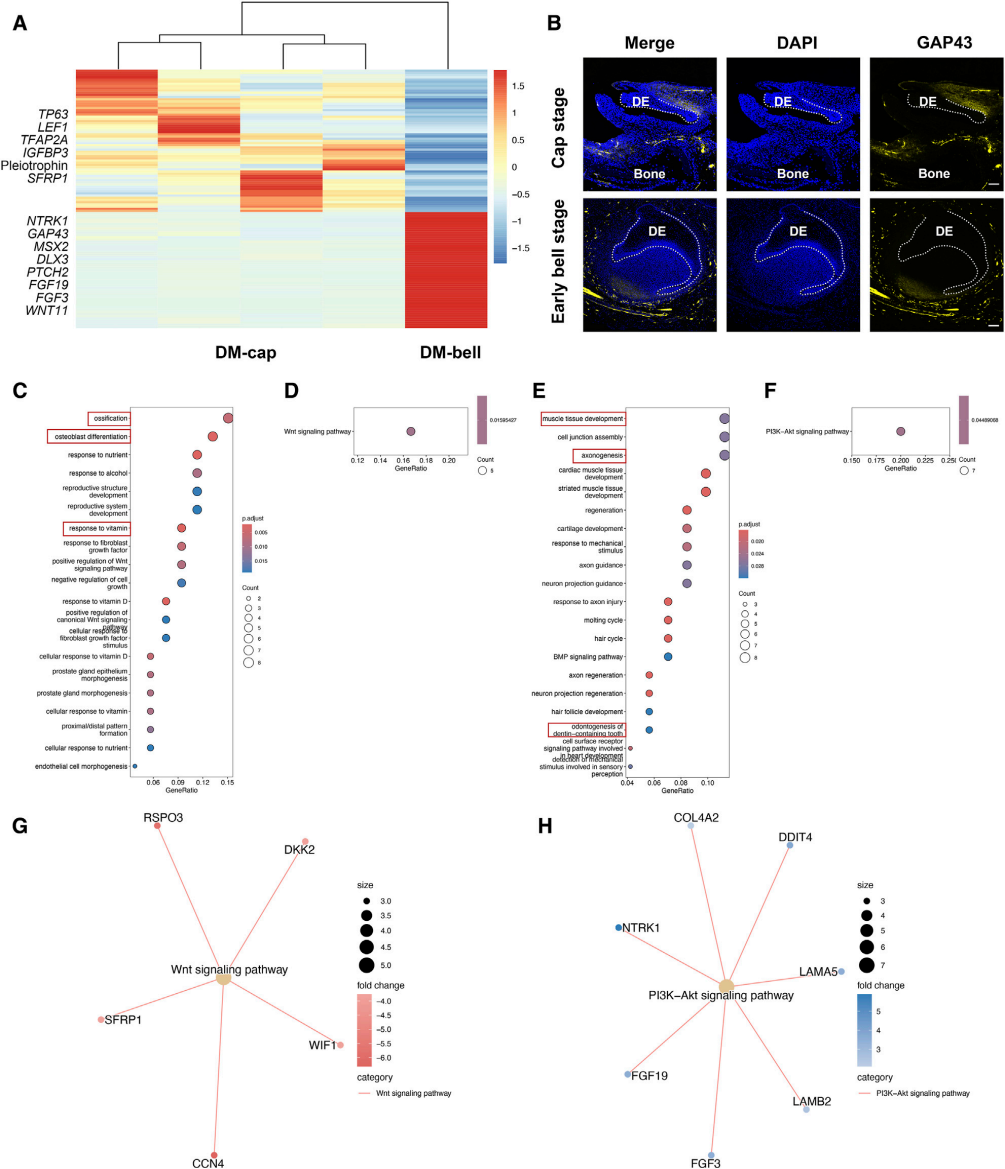
Temporal transcriptional changes between DM-cap and DM-bell. **(A)** Heatmap for DEGs identified in DM-cap and DM-bell. **(B)** Representative immunofluorescence staining images of GAP43 in DM-cap and DM-bell. The white line outlines dental epithelium. DE: dental epithelium. Scale bar: 50 μm (Cap stage), and 100 μm (Early bell stage). **(C)** GO analysis for DEGs upregulated in DM-cap. **(D,G)** KEGG pathway analysis for DEGs upregulated in DM-cap. **(E)** GO analysis for DEGs upregulated in DM-bell. **(F,H)** KEGG pathway analysis for DEGs upregulated in DM-bell.

Since dental mesenchyme obtained odontogenic potential at the early bell stage, we speculated that the molecular signatures in dental mesenchyme vary from the cap stage to the early bell stage. We then investigated the transcriptional changes of dental mesenchyme between these two different developmental stages. In total, we identified 66 DEGs at the cap stage and 73 DEGs at the early bell stage (Figure 4A). The biological processes involved in the dental mesenchyme at the cap stage (DM-cap) mainly encompassed ossification, osteoblast differentiation, and response to vitamin D, indicating intensive bone formation activities preceding odontogenesis in dental mesenchyme (Figure 4C).

In DM-bell, the upregulated genes, such as Neurotrophic Receptor Tyrosine Kinase 1 (*NTRK1*) and Growth Associated Protein 43 (*GAP43*) enriched in axonogenesis, *MSX2 and* Distal-Less Homeobox 3 (*DLX3*) enriched in odontogenesis of dentin-containing tooth (Figure 4E). Besides, we detected a large number of activities associated with ECM organization, such as muscle tissue development, cell junction assembly, and cardiac muscle tissue development (Zhang et al., 2021). To sum up, GO analysis of DE-cap, as well as DM-bell, is consistent with the results of the tooth germ recombination experiments conducted by Hu et al. (2014). Besides, GO analysis revealed strong axonogenesis activities in DM-bell as well. GAP43 is a nervous system-specific, growth-associated protein, which was listed as the top DEG in DM-bell. We identified a large number of nerves surrounding the tooth germ at the early bell stage, which were identified by the immunofluorescence staining of the neuronal marker Tubulin β III as well. Besides, we detected the specific expression of GAP43 at the root of the dental papilla in the dental mesenchyme (Figure 4B; Supplementary Figure S2).

Besides, the signaling pathways were different between DM-cap and DM-bell by KEGG pathway analysis, we found that the Wnt signaling pathway was activated at the cap stage, while it was replaced by the PI3K-Akt signaling pathway at the early bell stage (Figures 4D, F, G, H).

In this part, we observed that dental mesenchyme possessed osteogenic capacity at the cap stage before acquiring tooth-inductive potential. In DM-bell, newly-formed nerves tightly surrounded the tooth germ, appearing at the root of the dental papilla simultaneously. It is at the early bell stage that dental papilla obtains odontogenic capacity. We therefore hypothesized that axonogenesis might contribute to the odontogenic potential of the dental mesenchyme at the early bell stage.

### 3.4 Candidate genes involved in the shift of tooth-inductive capability

**FIGURE 5:**
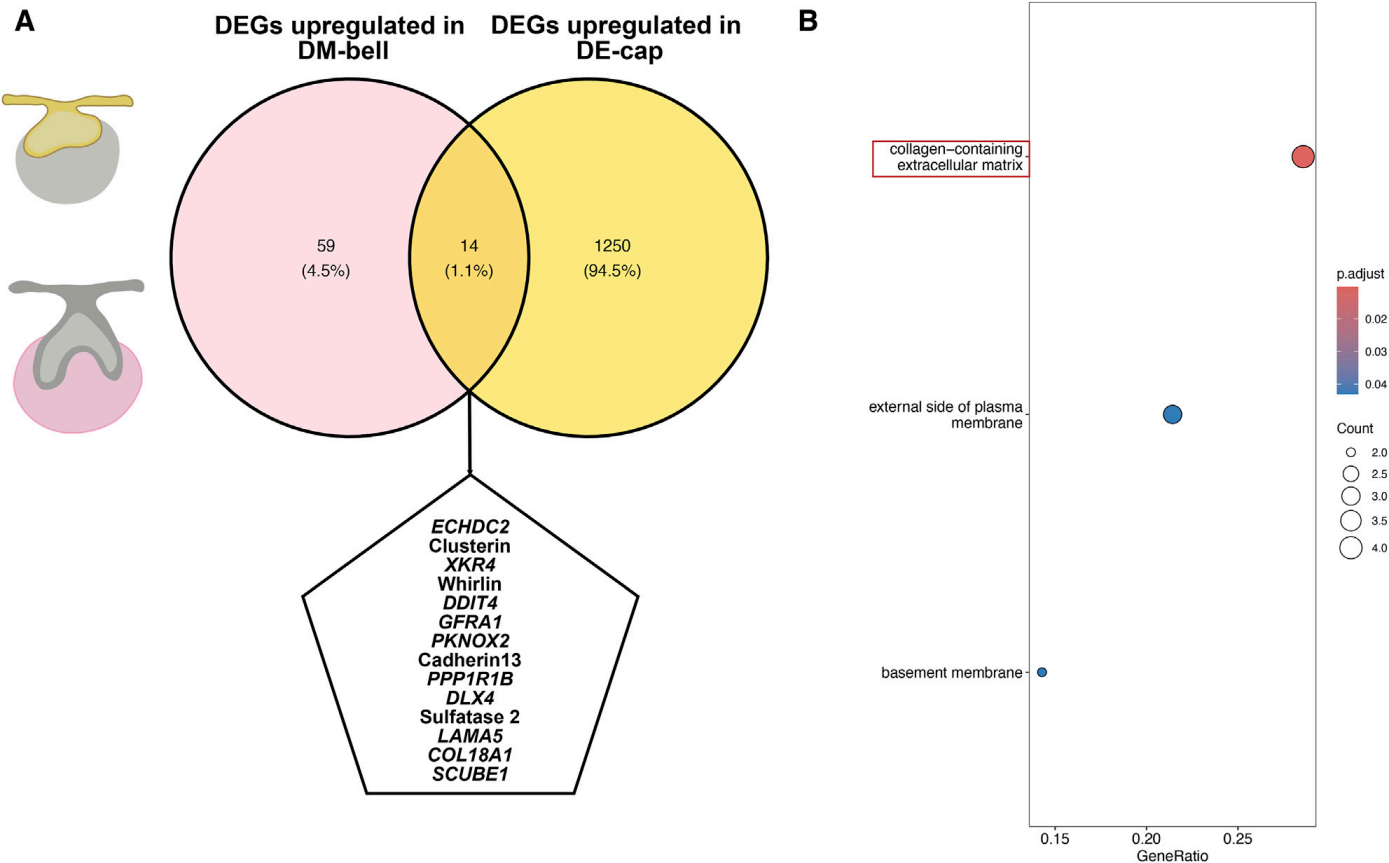
DEGs associated with the shift of tooth-inductive capability. **(A)** Venn graph for DEGs both upregulated in DE-cap and DM-bell. **(B)** KEGG pathway analysis for the shared DEGs.

To identify the crucial genes involved in the shift of tooth-inductive capability, we examined genes both upregulated in DE-cap and DM-bell. We identified 59 upregulated DEGs in DM-bell and 1,250 upregulated DEGs in DE-cap. In total, we found 14 shared genes in these two compartments, which might explain the mechanism of the odontogenic capacity shift occurs (Figure 5A). Genes like XK Related 4 (*XKR4*)*,* GDNF Family Receptor Alpha 1 (*GFRA1*)*,* Cadherin 13, Laminin Subunit Alpha 5 (*LAMA5*)*, COL18A1,* and Signal Peptide, CUB Domain And EGF Like Domain Containing 1 (*SCUBE1*) encodes cell surface proteins or ECM proteins. Enoyl-CoA Hydratase Domain Containing 2 (*ECHDC2*)*,* Protein Phosphatase 1 Regulatory Inhibitor Subunit 1B (*PPP1R1B*)*,* and Sulfatase 2 encodes proteins related to different kinds of enzymes. KEGG pathway analysis of these shared genes mostly enriched collagen-containing extracellular matrix (Figure 5B). This was similar to the study conducted by Chen et al. (2023) between dental epithelium at E11.5 and dental mesenchyme at E13.5 on a mouse model, as they found extracellular proteoglycans and their distinct sulfation played important roles in early odontogenesis. The Sulfatase 2 gene found in our study is an important paralog of the Sulfatase 1 gene found in the study conducted by Chen et al. (2023), which was considered a glycosaminoglycan biosynthetic enzyme involved in odontogenesis capacity shift. Sulfatase 1 and Sulfatase 2 enzymes modify Heparan sulfate proteoglycans sulfation by removing 6-O sulfates to regulate cell signaling (Okada et al., 2017). Taken together, we concluded that ECM and enzymes, especially sulfatase, might explain the mechanism of the shift of tooth-inductive capability.

### 3.5 Species comparisons to reveal hallmark events during the shift in odontogenic potential in both humans and mice

**FIGURE 6:**
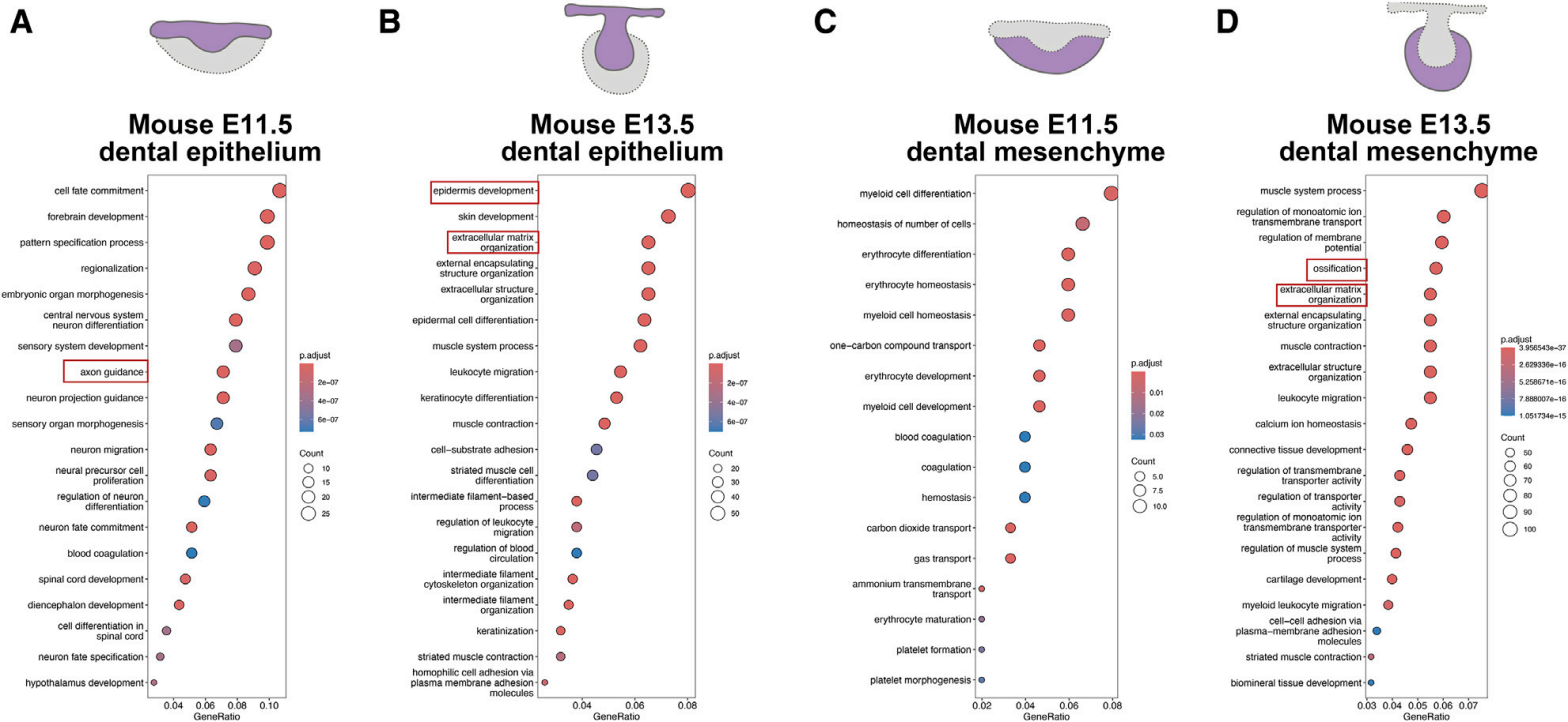
Odontogenesis process in mice (adapted from Chen et al., 2023). **(A)** GO analysis for DEGs upregulated in mouse E11.5 epithelium. **(B)** GO analysis for DEGs upregulated in mouse E13.5 epithelium. **(C)** GO analysis for DEGs upregulated in mouse E11.5 mesenchyme. **(D)** GO analysis for DEGs upregulated in mouse E13.5 mesenchyme.

In order to figure out a conserved core mechanism of odontogenesis, we further compare the odontogenesis process between humans and mice to identify their commonalities. We investigated the odontogenesis process in mice by using data from previous studies (Chen et al., 2023). On the mouse model, odontogenic potential initially resides in dental epithelium at E11.5, and then shifts to dental mesenchyme at E13.5 (Mina and Kollar, 1987). We compared transcriptional changes at different developmental stages in mouse dental epithelium and dental mesenchyme respectively. GO analysis revealed a large amount biological processes associated with axon guidance in mouse E11.5 epithelium (Figure 6A). The biological processes involved in mouse E13.5 epithelium were mainly epidermis development and ECM organization (Figure 6B). In mouse E11.5 mesenchyme, we enriched biological processes mostly related to erythrocytes, indicating extensive blood vessels formation in it (Figure 6C). We enriched ECM organization and ossification in mouse E13.5 mesenchyme (Figure 6D).

When comparing the biological processes involved in odontogenesis between humans and mice, we observed axon guidance, ECM organization, processes related to erythrocytes, and ossification were predominantly involved, albeit enriched in different compartments and developmental stages (Table 1). Therefore, we designated angiogenesis, ossification, axonogenesis, and ECM organization, as hallmark events in odontogenesis, collectively contributing to the process of tooth formation.

**TABLE 1:** Biological processes involved in dental epithelium and dental mesenchyme at different developmental stages between humans and mice.

		Humans	Mice
Stage 1^a^	Dental epithelium	odontogenesis epidermis development	axon guidance
	Dental mesenchyme	ossification	processes related to erythrocytes
Stage 2	Dental epithelium	processes related to erythrocytes	epidermis development ECM organization
	Dental mesenchyme	odontogenesis axon guidance ECM organization	ECM organization ossification

^a^ Stage 1: cap stage in human tooth; E11.5 in mouse tooth; Stage2: early bell stage in human tooth; E13.5 in mouse tooth.

## 4 Discussion

Studies on the developmental patterns and molecular mechanisms underlying odontogenesis serve as the foundation for regenerative strategies targeting dental tissues. In this study, we constructed spatiotemporal transcriptomic profiles of human deciduous tooth germs during the critical period of tooth formation by coupling LCM and RNA-seq. We provided bioinformatic evidence that DE-cap as well as DM-bell possessed tooth-inductive capability. Furthermore, we observed the formation of blood vessels and nerves in the tooth germs coinciding with the acquisition of odontogenic potential, as well as the presence of osteogenic capacity in dental mesenchyme preceding the acquisition of odontogenic potential. We examined the genes upregulated in both DE-cap and DM-bell, leading to the conclusion that ECM might explain the mechanisms involved in the shift of tooth-inductive capability. Species comparisons between humans and mice suggested that nerves, ECM, erythrocytes, and osteoblasts played essential roles in the odontogenesis process. Collectively, we identified angiogenesis, ossification, axonogenesis, and ECM organization as hallmark events in odontogenesis.

Reciprocal interactions between epithelium and mesenchyme are of great importance to the development of many organs, including hair follicles, mammary glands, salivary glands, and teeth (Jiménez-Rojo et al., 2012). During the crosstalk between the two compartments, the epithelium undergoes thickening and invagination into the mesenchyme, followed by condensation of the surrounding mesenchyme, ultimately leading to the formation of highly specialized structures. The odontogenic potential is crucial in odontogenesis as it plays a key role in instructing tooth formation. The tooth recombination assay conducted by Hu et al. (2014) revealed that odontogenic potential resides in the human dental epithelium at the cap stage and then shifts to the dental mesenchyme at the bell stage. Our LCM-seq results are consistent with the findings of Hu et al. (2014). In addition, our LCM-seq analysis suggested that the DM-cap possesses osteogenic potential. Although evidence in human samples is lacking, a study conducted in miniature pigs demonstrated this possibility. Researchers found that re-aggregation of dental mesenchymal cells isolated from the cap stage formed bone tissues after 8 weeks of subrenal capsule transplantation (Wang et al., 2018). Moreover, according to the trajectory analysis of the first pharyngeal arch of mice (Yuan et al., 2020), both odontogenic lineage and osteogenic lineage originate from cranial neural crest (CNC)-derived progenitors, which acquire their fates through an orderly sequential bifurcation process. Odontogenic lineage and osteogenic lineage share a common differentiation pathway at the first bifurcation fate decision, while the second bifurcation separates the odontogenic lineage from the osteogenic lineage. This aligns with our conclusion and confirms the close relationship between osteogenic and odontogenic activities.

Both angiogenesis and axonogenesis are considered as crucial activities in organogenesis and regeneration (Crivellato, 2011; Sinigaglia and Averof, 2019). In addition to providing oxygen and nutrients, blood vessels can also provide regulatory signals to instruct surrounding cells during the development process (Crivellato, 2011). Blood vessels surrounding the outer enamel epithelium, as well as in the dental papilla, were identified at the early bell stage through immunofluorescence staining of VEGF in human tooth germs. VEGF is a secreted protein that acts as an important regulator of blood vessel formation. Shadad et al. (2019) observed the spatiotemporal expression of *Vegf* and *Vegfr2* in the developing mouse molars. Thus, the newly formed blood vessels provide blood supply to the developing tooth germ. Additionally, VEGF secreted by endothelial cells likely participates in the odontogenic differentiation process. Nerves contribute to organogenesis in similar ways, serving as a scaffold for other cells with important roles or as a source of instructive signals (Sinigaglia and Averof, 2019). Intensive nerve formation was detected at the early bell stage in human tooth germs through immunofluorescence staining of GAP43. LCM-seq also revealed the gene expression of molecules such as *FGF19* and *FGF3* secreted by nerves, potentially serving as instructive signals contributing to tooth formation. Sensory nerves are a vital component of the dental pulp, crucial for tooth formation, homeostasis, and repair. Pei et al. (2024) used a mouse molar root development model to investigate the role of sensory nerves in organ formation, revealing that sensory nerves influence root development by regulating progenitor cell fate through FGF-SHH signaling. Since GAP43 is specifically expressed in the apical papilla of human tooth germs, our finding also suggests the critical role of sensory nerves in human tooth root formation. Additionally, sensory nerves specifically expressed in the dental follicle might also play a crucial role in the development of periodontal tissues.

The ECM is a complex network of proteins and carbohydrates that provides structural and biochemical support to the surrounding cells, playing a crucial role in cell adhesion, migration, differentiation, and signaling (Faulk et al., 2014). We identified 14 shared genes regulated in DE-cap and DM-bell, indicating the crucial genes involved in odontogenesis. KEGG pathway analysis revealed that ECM played a crucial role in the shift of odontogenic potential. Similarly, a previous study conducted on mice demonstrated the important roles of extracellular proteoglycans and their biosynthetic enzymes in odontogenesis. They found the ECM exerted a profound influence on odontogenesis by providing structural support, guiding cell behavior, regulating differentiation, and participating in signaling events (Chen et al., 2023). Our findings offered novel evidence in support of this viewpoint within the context of human embryonic tissues.

Species comparisons between humans and mice revealed several shared biological processes during tooth formation, including axon guidance, ECM organization, processes related to erythrocytes, and ossification. Although these shared processes occurred in different compartments at different developmental stages, they suggested the essential biological processes involved in odontogenesis. The study conducted by Chen et al. (2023) included both mouse incisors and molars. Although mouse incisors exhibit lifelong growth, which might differ significantly from human tooth development, we believe that studying various types of teeth across different species can provide a more comprehensive understanding of tooth development mechanisms. Collectively, we deduced that tooth formation is a complex process involving the participation of nerves, blood vessels, osteoblasts, and ECM to orchestrate osteogenesis toward odontogenesis.

However, late-stage embryos suitable for collection are rare in clinical practice, especially those requiring voluntary termination of pregnancy through surgical procedures, resulting in our inability to collect enough tooth germs at the early bell stage. Moreover, due to challenges in accurately identifying specific tooth types in early embryo mandibles and compounded by insufficient numbers of human tooth germs, we conducted analyses pooling all types of tooth germs together in this study. For future studies, we aim to collect more diverse samples of tooth germs and compare the development of different types to elucidate the mechanisms underlying tooth development more clearly.

In conclusion, this study unveiled transcriptomic profiles of human dental epithelium and mesenchyme during the critical period of odontogenesis, identifying several hallmark events, including angiogenesis, ossification, axonogenesis, and extracellular matrix (ECM) organization. These findings provide insights into the crucial mechanisms involved in odontogenesis.

## Data Availability

The datasets presented in this study can be found in online repositories. The names of the repository/repositories and accession number(s) can be found below: https://www.ncbi.nlm.nih.gov/, PRJNA1067279.

## Supplementary material

SUPPLEMENTARY FIGURE S1 Representative immunofluorescence staining images of CD31 in DE-cap and DE-bell. The white line outlines dental epithelium. DE: dental epithelium. Scale bar: 50 μm.

SUPPLEMENTARY FIGURE S2 Representative immunofluorescence staining images of Tubulin β III in DM-cap and DM-bell. The white line outlines dental epithelium. DE: dental epithelium. Scale bar: 50 μm (Cap stage), and 100 μm (Early bell stage).
