# Supplementary figures and images for: Spatial and temporal gene expression patterns during early human odontogenesis process
Source: Front Bioeng Biotechnol. 2024 Jul 16;12:1437426. doi: 10.3389/fbioe.2024.1437426 (PMC11287127; doi:10.3389/fbioe.2024.1437426)

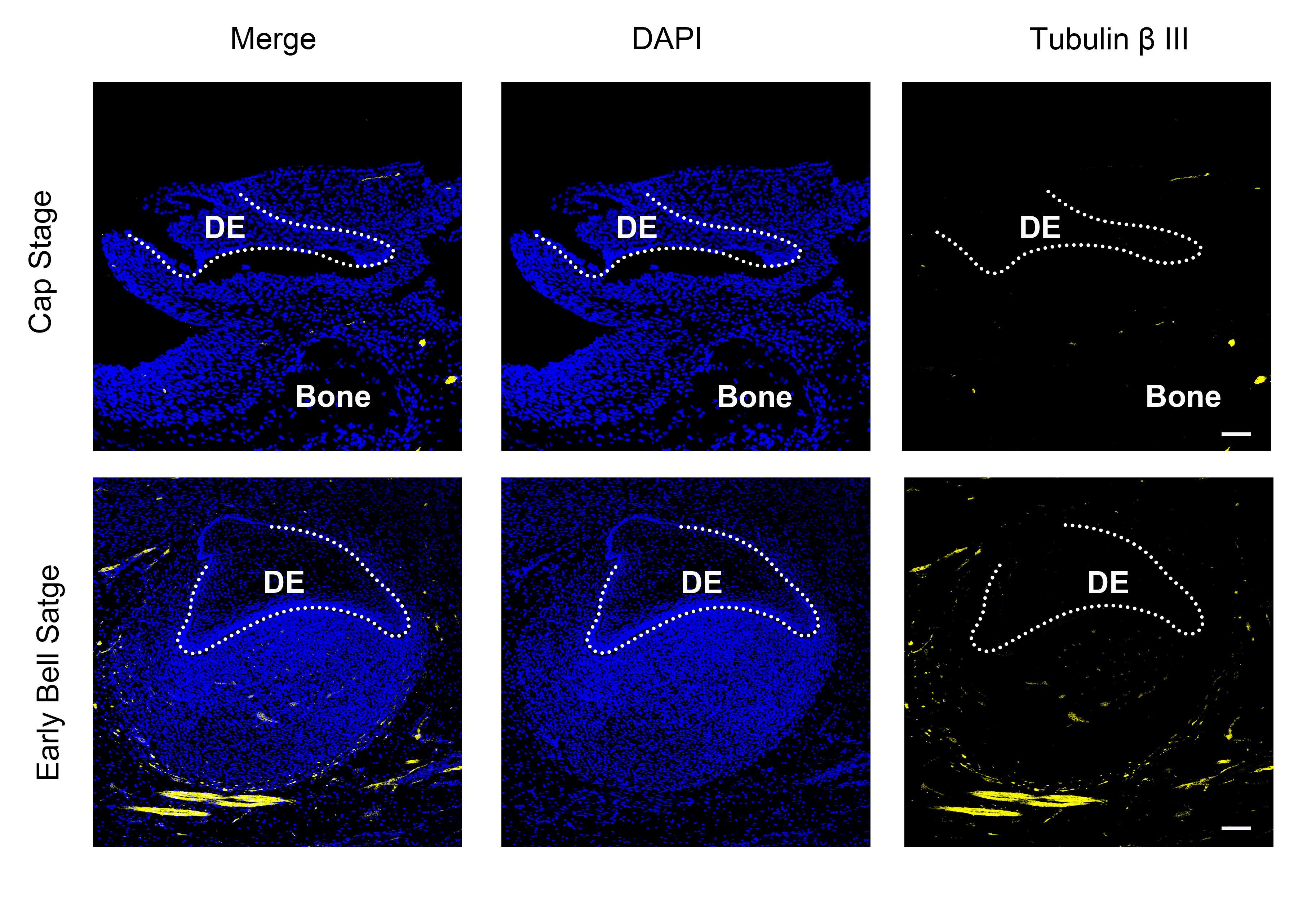

Supplement: Supplementary file 2 [file Image2.TIF]

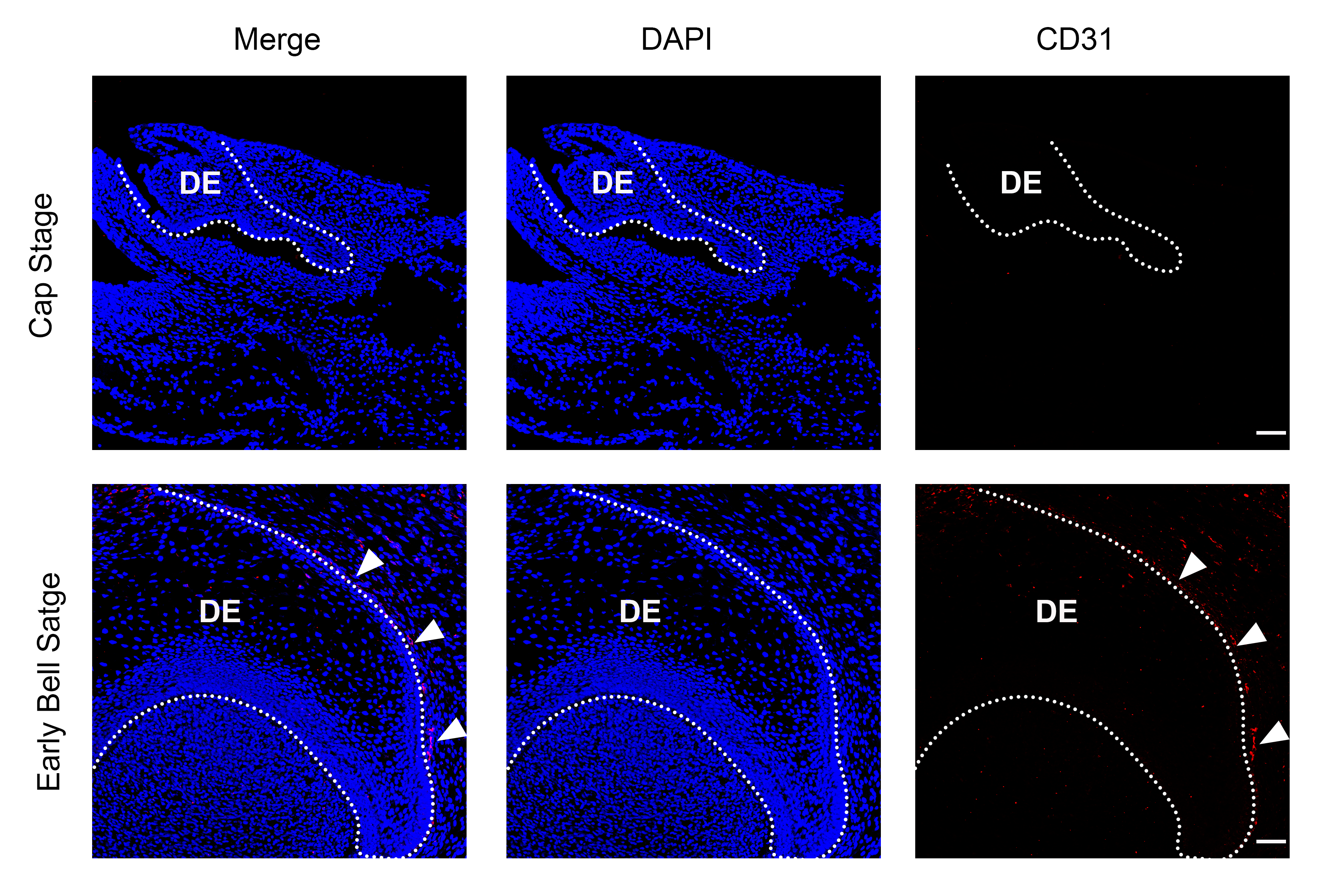

Supplement: Supplementary file 3 [file Image1.TIF]
